# Scoping the impact of the national child measurement programme feedback on the child obesity pathway: study protocol

**DOI:** 10.1186/1471-2458-12-783

**Published:** 2012-09-13

**Authors:** Catherine Falconer, MinHae Park, Áine Skow, James Black, Ulla Sovio, Sonia Saxena, Anthony Kessel, Helen Croker, Steve Morris, Russell Viner, Sanjay Kinra

**Affiliations:** 1 Department of Non-Communicable Disease Epidemiology, London School of Hygiene and Tropical Medicine, London, UK; 2 Department of Primary Care and Public Health, Imperial College, London, UK; 3 Faculty of Public Health and Policy, London School of Hygiene and Tropical Medicine, London, UK; 4 Health Behaviour Research Centre, University College London, London, UK; 5 UCL Research Department of Epidemiology and Public Health, University College London, London, UK; 6 General and Adolescent Paediatrics Unit, Institute of Child Health University College London, London, UK

**Keywords:** Childhood obesity, National Child Measurement Programme

## Abstract

**Background:**

The National Child Measurement Programme was established to measure the height and weight of children at primary school in England and provides parents with feedback about their child’s weight status. In this study we will evaluate the impact of the National Child Measurement Programme feedback on parental risk perceptions of overweight, lifestyle behaviour and health service use.

**Methods:**

The study will be a prospective cohort study of parents of children enrolled in the National Child Measurement Programme and key service providers from 5 primary care trusts (administrative bodies responsible for providing primary and secondary care services). We will conduct baseline questionnaires, followed by provision of weight feedback and 3 follow up questionnaires over the course of a year. Questionnaires will measure change in parental risk perception of overweight, health behaviours and health service use. Qualitative interviews will be used to identify barriers and facilitators to change. This study will produce preliminary data on National Health Service costs associated with weight feedback and determine which feedback approach (letter and letter plus telephone) is more effective.

**Discussion:**

This study will provide the first large scale evaluation of the National Child Measurement Programme feedback. Findings from this evaluation will inform future planning of the National Child Measurement Programme.

## Background

The prevalence of overweight and obesity in children and young people has more than tripled since the 1980s, and is now a major public health concern for the United Kingdom (UK) [1]. The National Child Measurement Programme (NCMP) for England was established by the Department of Health in 2005 to measure the height and weight of every child in reception (aged 4–5 years) and year 6 (aged 10–11 years) at state primary schools in England [2]. The findings of the NCMP are used to gather population surveillance data and to inform local planning of services for children and young people. The most recent NCMP report suggests that 22.6% of children in reception year and 33.4% in year 6 are either overweight or obese, defined using the UK 1990 growth chart cut offs [3,4].

Since 2008, the NCMP guidance to Primary Care Trusts (PCTs) has encouraged provision of routine feedback to parents regarding their child’s weight status. The aims of the feedback are to increase public understanding of child weight issues and assist families to make healthy lifestyle choices. There is evidence that parents have a poor recognition of their child’s weight status [5-7] and parents who are unable to identify their child’s overweight and the associated health risks may be less likely to prioritise promoting healthy lifestyle behaviours and seeking help for their child [8,9]. The provision of feedback on weight may improve parents’ ability to recognise overweight in their children, and prompt changes in health behaviours [5]. Feedback is usually provided as a letter, but proactive feedback in the form of an additional telephone call to parents of children who fall outside the ‘healthy weight’ range is also encouraged. A pilot study of routine NCMP feedback suggested the majority of parents (over 90%) find the feedback letter to be helpful [10]. However, to date there has been no large scale evaluation of the effectiveness and acceptability of feedback.

The specific objectives of the study are to estimate the impact of NCMP feedback on parents’ perceptions of the health risks associated with their child’s overweight, behaviour change and health service use, and to identify barriers and levers to behaviour change and health service use. The study will also produce preliminary data on National Health Service (NHS) costs associated with NCMP feedback and will try to determine which feedback approach (letter versus letter plus proactive) has the greater positive impact.

## Methods

### Setting

We will recruit five primary care trusts (PCTs), administrative bodies responsible for providing primary and secondary care services, from in and around London and invite all parents of children enrolled in the NCMP to participate.

### Design

This study will establish a prospective cohort of parents of children enrolled in the NCMP and will use a mixed methods approach.

#### Outcomes

The primary outcome will be the change in parental perception of the health risks associated with a child’s overweight 1 month after receiving weight feedback

Secondary outcomes of interest will be:

1. Change in parental perception of child’s weight status 1 month after receiving weight feedback.

2. Lifestyle behaviours (diet, physical activity and sedentary behaviour),

3. Health service use: attendance and frequency of attendance at locally available services,

4. Experience of the feedback process, including acceptability of the routine feedback, assessed 1 month after feedback.

### Time line

**Figure 1:**
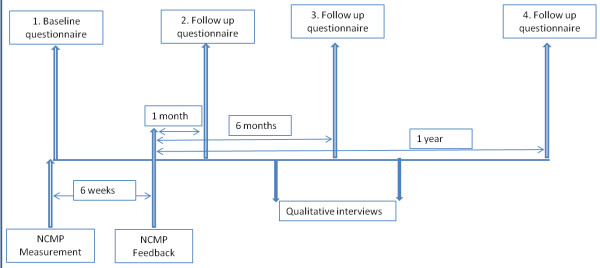
**Anticipated time line of the study** .

The anticipated time line for the study is outlined in Figure 1.

#### Data collection: Questionnaire surveys

Questionnaires will be developed to collect information on a range of socio-demographic variables, lifestyle behaviours, parental perceptions and health service use, using previously used or validated questionnaires where possible. A short form questionnaire will also be developed to capture the key outcomes and demographic variables from non-respondents.

##### Baseline survey

We will distribute the baseline questionnaire through schools to all children enrolled in the NCMP within the recruited PCTs on the day of the NCMP measurement. The questionnaire will also be made available online and the short form of the questionnaire will be distributed to all non-responders. All schools will be encouraged to distribute posters and postcards to promote participation.

Baseline questionnaires will provide a detailed description of the study, therefore informed consent will be assumed for all participants who return completed questionnaires. Parents will be asked to provide contact details for future correspondence. The child will be allocated a unique identification number which will be used for all future correspondence. The contact details page will be removed from the questionnaire and stored separately in a locked filing cabinet.

Data on children’s height and weight will be obtained directly from PCTs. All NCMP measurements will be uploaded by PCT staff onto a tool provided by the NHS Information Centre for Health and Social Care which calculates the child’s BMI centile and category based on their height and weight measurements, age and gender. We will match children participating in the study to their NCMP data (height, weight, BMI, BMI category and date of measurement) in the PCT database using a macro designed in Microsoft Access which matches on name and date of birth. Children who cannot be automatically matched will be identified manually.

De-identified data for all children enrolled in the PCT will be used to provide general demographic information for the entire population. The following variables will be requested for all children: BMI (with height and weight), ethnicity, date of birth, age at measurement, gender, and postcode (converted to an Index of Multiple Deprivation (IMD) score). Information that could potentially allow identification of individuals who have not agreed to participate in the study will not be removed from the PCT.

##### Follow up surveys

Follow-up surveys will be distributed 1 month, 6 months and one year after parents receive NCMP feedback. We will send reminder short form follow up questionnaires to all who fail to respond within two weeks, and also send email links to online surveys, text message reminders and reminder phone calls. All parents will receive at least two reminders.

### Data collection: Qualitative research

#### Parent interviews

Individual interviews will be performed with the parents and families of 50 children classified by the NCMP as ‘overweight’ or ‘very overweight’. We will select children on the basis of the primary outcome, *i.e.* change in risk perception following NCMP feedback. The sample will be selected so that 50% of children will be from deprived/ethnic minority households. Interviews will be completed within the participant’s home. The interviews will explore barriers and levers to change using thematic analyses [11] to interpret the responses.

#### Staff interviews

A small number (n = 15) of interviews will be carried out with staff from each of the PCTs; staff will be purposively selected to capture the main provider groups (schools and practice nurses, community weight management services, PCT obesity leads, *etc.*). The interviews will be used to explore providers’ views on the provision of the NCMP and obesity services.

### Analysis

#### Quantitative analysis

The demographic characteristics of study respondents will be described, and compared with those of non-responders.

Univariable analysis will test for associations between the primary outcome and explanatory variables. Multivariable logistic regression models will be fitted to the data to identify predictors of change in parental risk perception, lifestyle behaviour and help seeking. Analyses will be performed on the whole study sample and in a sub-sample composed only of overweight and very overweight children. Data will initially be analysed separately for each age-PCT group, and may be combined in analyses accounting for clustering where appropriate. Proportions will be calculated to summarise the parental experience of the feedback process, differences between groups according to the BMI feedback received (letter feedback compared to letter plus proactive telephone feedback) and child’s weight status (healthy weight compared to overweight or very overweight).

#### Qualitative analysis

All individual interviews will be transcribed and NVivo software (QSR International) will be used to conduct thematic analyses. Each transcript will be read and re-read in order to familiarise the researcher with the data, and key ideas and recurrent themes will be identified.

#### Power and sample size

We have powered our study to detect an effect size (defined as the proportion of parents changing their perception of health risks of their child’s overweight) of 5%. If 30% of parents of overweight and very overweight children at baseline are able to identify the health risks of their child’s weight, a sample size of 800 will provide 80% power to detect an effect size of 5% at p < 0.05 statistical significance level. If the proportion of parents able to identify the risk is 10% at baseline, a sample size of 800 will provide a power of 100% (Table 1).

**Table 1:** Power (probability of detecting a true effect %) under a range of baseline proportions of parental awareness of the health risk of their child’s weight using a matched case–control design with a two sided alpha of 5% (calculations carried out using the sampsi_mcc command in STATA v10)

**Sample size**	**Effect size**	**Power (if 30% recognise health risk at baseline)**	**Power (if 10% recognise health risk at baseline)**
**200**	5%	28	58
**400**	5%	50	91
**800**	5%	80	100

A response rate of 30% is expected therefore an initial sample of 18,000 parents should provide at least 800 parents of overweight and very overweight children, allowing for attrition between surveys.

### Economic evaluation

We will use data from the PCTs to compile estimates of the cost of providing the two types of routine feedback (letter and letter plus proactive). PCTs will be asked to provide information on the contribution of staff and the resources required for delivering routine weight feedback. The cost of staff time will be evaluated using the NHS Agenda for Change 2012 pay scales (http://www.nhscareers.nhs.uk) and added to the estimated resource costs to provide a total cost for each PCT. The total cost will be divided by the number of children enrolled in the NCMP within each PCT and presented as a cost per child. Costs for providing each type of feedback will be averaged across all three areas to provide an average cost for the UK child. The relative costs of providing letter and proactive feedback will be compared and discussed with respect to their relative effectiveness.

### Ethical approval

This study has obtained full ethical approval for the study from the London School of Hygiene and Tropical Medicine (LSHTM) ethics committee. Within all PCTs, approval for the research to take place will be granted by the Director of Public Health.

## Discussion

This study will provide the first large scale evaluation of the impact of NCMP feedback on parental risk perceptions, health behaviours and help seeking behaviours. Data from the demographically and ethnically diverse cohort of parents will provide information on the effectiveness and relative costs of different methods of providing feedback, as well as socio-demographic influences on the impact of NCMP feedback. Previous weight feedback studies have generally focussed on outcomes of parental awareness of child overweight and potential distress of the child or parent [5,12]; few have assessed intention to change or actual changes in lifestyle behaviour. Previous studies have evaluated the impact of written feedback (including personalised health report cards and individually tailored feedback letters); however, to date there has been no evaluation of providing weight feedback *via* the telephone. The NCMP is a Department of Health Initiative and is conducted in all state primary schools around England. A few small scale evaluations of aspects of the NCMP feedback approach have been conducted; however, this study will offer the first formal and comprehensive evaluation of its impact [10]. The present study will examine multiple outcomes that are pertinent to the NCMP, including health service use and cost implications, enabling a more comprehensive evaluation of its impact.

The universal scope of the NCMP means that there is no comparison group against which the impact of feedback can be compared. In order to minimise some of the biases associated with pre-/post-test designs with a single group, a short time interval between baseline and follow-up questionnaires is planned; this will minimise the effect of any background behaviour changes that may occur independently of the feedback letter. Study instruments have also been designed to be as similar as possible at all time points. The use of self-report measures of lifestyle behaviour and health service use will increase the potential for recall bias; previously validated measures will be used where possible to minimise this bias.

It is anticipated that there may be a low response rate and response bias towards parents who are more interested and engaged in their child’s health [13]. To address these issues the questionnaire will be distributed to all parents of children enrolled in the NCMP and a number of reminders and incentives will be used. The study information provided to parents will highlight that the evaluation is being performed independently of the organisations responsible for the NCMP and that all views of the NCMP, whether positive or negative, will be welcomed. Questionnaires will be piloted with parents of school aged children to ensure the language used is easily understood and non-judgemental.

To summarise, this study will be the largest and most comprehensive evaluation of the NCMP feedback to date, with outcomes that cover a wide range of potential benefits or harms of receiving weight feedback.

## Abbreviations

BMI: Body Mass Index; NCMP: National Child Measurement Programme; LSHTM: London School of Hygiene and Tropical Medicine; PCTs: Primary Care Trusts; NHS: National Health Service; IMD: Index of Multiple Deprivation; NIHR: National Institute for Health Research.

## Competing interests

AK is also Director of Public Health Strategy and Medical Director at the Health Protection Agency (HPA). The views expressed here, however, are personal and are not intended to represent the views of the HPA.

## Authors’ contributions

The study was conceptualised by SK and RV. All authors read and approved the final manuscript.

## Pre-publication history

The pre-publication history for this paper can be accessed here:

http://www.biomedcentral.com/1471-2458/12/783/prepub
